# Development of a UHPLC-MS/MS Method for the Determination of Moxidectin in Rat Plasma and Its Application in Pharmacokinetics

**DOI:** 10.3390/molecules29204786

**Published:** 2024-10-10

**Authors:** Hongjuan Zhang, Zhen Yang, Baocheng Hao, Di Wu, Dan Shao, Yu Liu, Wanxia Pu, Shouli Yi, Ruofeng Shang, Shengyi Wang

**Affiliations:** Key Laboratory of New Animal Drug Project, Gansu Province/Key Laboratory of Veterinary Pharmaceutical Development, Ministry of Agriculture and Rural Affairs/Lanzhou Institute of Husbandry and Pharmaceutical Sciences of CAAS, Lanzhou 730050, China; zhanghongjuan@caas.cn (H.Z.);

**Keywords:** UPLC-MS/MS, pharmacokinetics, moxidectin, rat, microspheres

## Abstract

The aim of the present study was to establish a simple and reliable ultra-high-performance liquid chromatography tandem mass spectrometry (UHPLC-MS/MS) method and apply it for the determination of pharmacokinetics of moxidectin-loaded microspheres (MOX-MS) in rats. Plasma samples were processed using a simplified liquid–liquid extraction method and were separated using an Agilent Zorbax Eclipse Plus C18 column (50 mm × 2.1 mm, 1.8 μm) with a mobile phase consisting of a 10 mM ammonium formate solution with 0.1% formic acid (A) and acetonitrile (B) at a flow rate of 0.4 mL/min for 5 min. Avermectin B1a was used as an internal standard (IS). The sample was injected at a volume of 10 μL with a column temperature of 35 °C and detected in a positive ion mode. A good linear response across the concentration range of 1.00–200 ng/mL (r^2^ > 0.99) and a lower limit of quantification (LLOQ) of 1.00 ng/mL were achieved. The extraction recovery of moxidectin exceeded 94.1%, the matrix effect was between 91.2% and 96.2%, the accuracy ranged from 100.1 to 103.6%, and the relative standard deviation (RSD) did not exceed 15% for the intra- and inter-day accuracy and precision. The pharmacokinetic results showed that MOX-MS significantly decreased C_max_, prolonged T_1/2_, and improved bioavailability. The developed method significantly reduced the assay volume, shortened detection time, simplified sample processing methods and saved assay costs, which may contribute to the development of the new antiparasitic drug.

## 1. Introduction

Moxidectin is a novel second-generation semi-synthetic macrolide drug that belongs to the milbemycin chemical family and is widely used clinically for human use to treat onchocerciasis (river blindness) and for companion and food-producing animals to control internal and external parasites [1,2,3]. The primary antiparasitic mechanism of moxidectin is binding to gamma-aminobutyric (GABA) and glutamate-gated chloride channels, causing hyperpolarization in nerves and muscle fibers, which paralyzes the organism, leading to its death. Glutamate channels are only expressed in invertebrates but not in mammalian hosts, making it relatively safe for mammals to use moxidectin [4]. In addition, moxidectin has many advantages over other macrocyclic lactones, including a single component, a broad antiparasitic spectrum, a long half-life, a high potency against parasites, and a great margin of safety [5,6].

Because of its excellent physicochemical properties, moxidectin has been developed into many dosage forms, including tablet, injection, pour-on, oral drench, and oral gel for the prevention of endo- and ecto-parasites in dogs, cattle, sheep, pigs and horses (Simparica Trio^®^, Proheart^®^, Cydectin^®^, Equest^®^ and Quest^®^) [4,7,8]. At present, there are only conventional preparations of moxidectin in China, which require frequent long-term administration. In order to meet the clinical needs of companion animals for the long-term prevention and treatment of parasitic disease, our group is developing a sustained-release microsphere injection of moxidectin (MOX-MS), which is designed to reduce the frequency of drug administration, improve animal compliance, and achieve better therapeutic effects and drug resistance management [9,10,11]. Conducting pharmacokinetic tests of MOX-MS is critical to understanding its in vivo sustained-release effect and predicting its efficacy. Thus, it is imperative to develop a simple, convenient, and sensitive method for the determination of the drug in plasma to accelerate the development of MOX-MS.

So far, the pharmacokinetics of moxidectin have been studied in various animals following various routes of administration [6,12,13,14,15]. Currently reported methods for detecting moxidectin in biological samples include high-performance liquid chromatography with fluorescence detection (HPLC-FD) and ultra-high-performance liquid chromatography tandem mass spectrometry (UHPLC-MS/MS). The HPLC-FD method displayed high sensitivity and a low limit of detection (LOD) of 0.08 ng/mL, but the sample processing steps were cumbersome and time-consuming, requiring solid-phase extraction cartridges for sample purification and a derivatization step before detection [16,17,18]. The UHPLC-MS/MS method featured high sensitivity, a short detection time, and simple sample preparation. However, to our knowledge, the reported UHPLC-MS/MS methods either required a large amount of plasma (≥120 μL) and a long detection time (>5 min), or expensive purification processing (Solid phase extraction cartridges) [15,19]. Buchter et al. conducted pharmacokinetic studies of moxidectin in rats using UHPLC-MS/MS. But the disadvantage of this method was that an expensive microsampling device, Mitra^®^, was used in this study, and the LOD was up to 10 ng/mL [15].

In this experiment, a novel UHPLC-MS/MS method was developed and validated to determine the pharmacokinetics of MOX-MS in rats. The established method has the advantages of simple and inexpensive plasma sample pretreatment, a shortened detection time, and reduced plasma volume, making this UHPLC-MS/MS method more robust and efficient than the existing detection methods. In addition, the developed method was applied to the study of the pharmacokinetics of MOX-MS in rats, which will accelerate the development of new drugs.

## 2. Result

### 2.1. Mass Spectrometry and Liquid Chromatography

**Figure 1:**
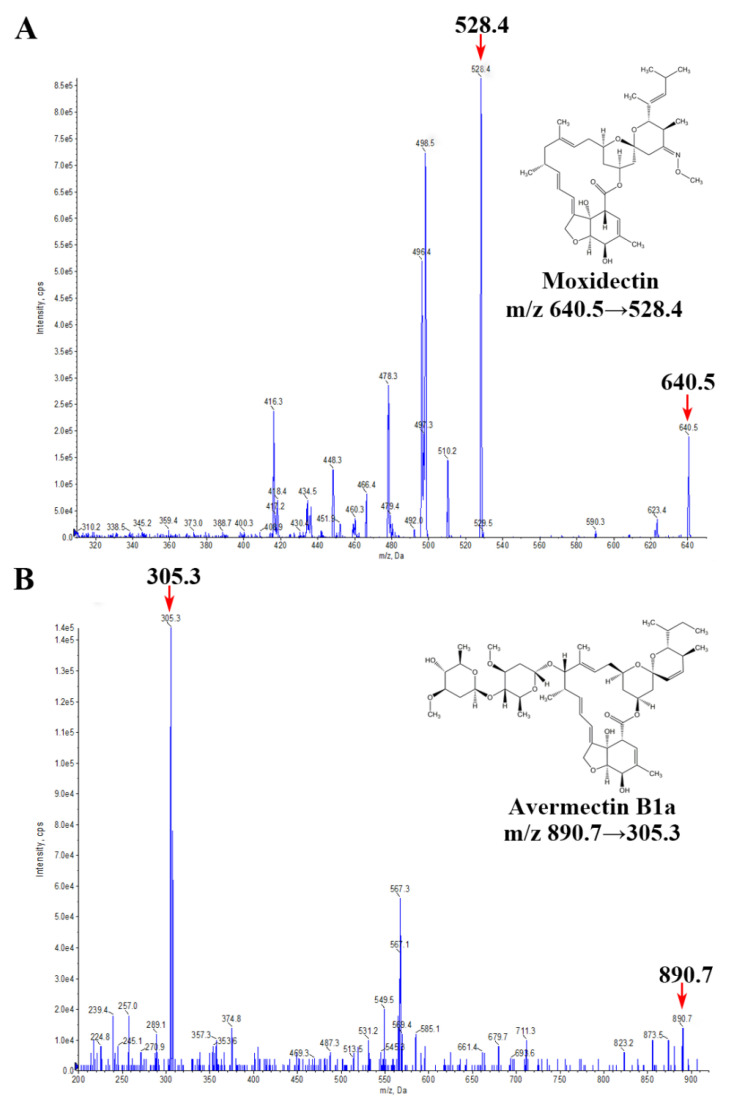
Product ion spectra of moxidectin (**A**) and avermectin B1a (**B**).

The positive ESI mode was chosen for MS detection as better MS responses of moxidectin and IS were achieved than those in the negative ESI mode. The product ion spectra of moxidectin and IS showed that the most abundant ions of moxidectin and IS were *m*/*z* 640.5 and *m*/*z* 890.7, respectively (Figures S1 and S2). The selected ion transitions of MRM for moxidectin and IS ranged from *m*/*z* 640.5 (Q1) to 528.4 (Q3) and *m*/*z* 890.7 (Q1) to 305.3 (Q3), respectively (Figure 1).

The chromatographic separation was carried out using an Agilent Zorbax Eclipse Plus C18 column (50 mm × 2.1 mm, 1.8 μm). Mobile phase A (10 mM ammonium formate solution with 0.1% formic acid) and mobile phase B (acetonitrile) with a ratio of 10/90 were used to isocratically elute the sample at a flow rate of 0.4 mL/min for 5 min. The retention times of moxidectin and IS were 2.24 min and 1.88 min, respectively. There were no endogenous substances in the rat plasma that interfered with the detection of analytes.

### 2.2. UHPLC-MS/MS Method Validation 

#### 2.2.1. Selectivity and Matrix Effects 

**Figure 2:**
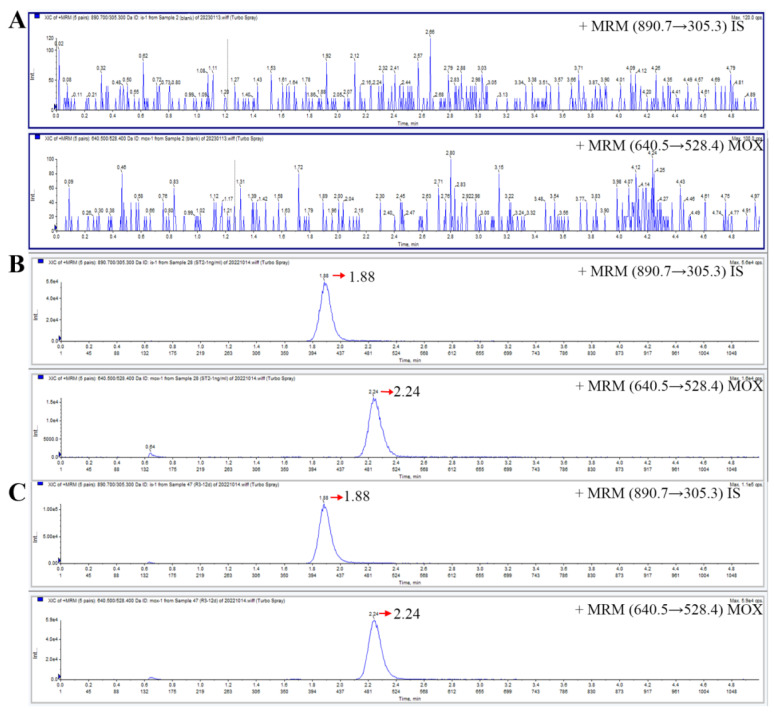
Chromatograms of moxidectin and IS in rat plasma. (**A**) blank plasma; (**B**) a blank plasma spiked with LLOQ; (**C**) a plasma sample obtained after a single subcutaneous injection of 1 mg/kg moxidectin.

The selectivity of the established method was evaluated by analyzing blank plasma samples from six different rats and the corresponding spiked plasma. Representative chromatographs of the blank plasma, blank plasma samples spiked with moxidectin and IS, and plasma samples from rats after subcutaneous administration are shown in Figure 2. There were no obvious interfering peaks around the retention times of moxidectin and IS. The chromatographic peaks of moxidectin and IS could be completely isolated from each another. These results indicated that endogenous substances did not interfere with the detection of moxidectin and IS.

The effect of the rat plasma matrix on moxidectin and IS was evaluated at three QC levels ranging from 91.2% to 96.2% (Table 1). These results showed that the rat plasma matrix did not significantly interfere with the detection of the established method.

#### 2.2.2. LLOQ and Linearity

The LLOD and the LLOQ of moxidectin were 0.200 ng/mL and 1.00 ng/mL, respectively. The calibration curve of moxidectin ranging from 1.00 to 200 ng/mL was calculated by the linear regression of the peak area ratio of moxidectin and IS and the concentration ratio of moxidectin and IS (Table 2). The calibration curve showed excellent linearity (r^2^ > 0.99) for the quantitative analysis of moxidectin in rat plasma.

#### 2.2.3. Accuracy and Precision

The accuracy and precision of the established method were evaluated at four QC levels (LLOQ, LQC, MQC and HQC). The intra- and inter-day accuracy of the method was 100.3–103.6% and 100.1–103.0%, respectively (Table 3). The intra- and inter-day precision of the method was 2.6–7.2% and 4.4–6.4%, respectively. All these results met the FDA’s criteria for bioanalytical method validation, suggesting that the established method was accurate and reproducible for the quantification of moxidectin in rat plasma.

#### 2.2.4. Recovery

The recovery of moxidectin in rat plasma was evaluated at three QC levels (LQC, MQC, and HQC). The average extraction recoveries of moxidectin at concentrations of 5.00 ng/mL, 50.0 ng/mL, and 150 ng/mL were 94.4 ± 3.8%, 94.1 ± 4.8%, and 98.0 ± 4.7%, respectively (Table 1).

#### 2.2.5. Stability

The stability of moxidectin in rat plasma was examined at three QC levels (LQC, MQC, and HQC) in different storage conditions. The accuracy and precision were 98.3–105.7% and 0.4–4.6%, respectively (Table 4), indicating that moxidectin was stable under the above tested storage conditions.

#### 2.2.6. Dilution Integrity

The dilution integrity of moxidectin in rat plasma was studied since the plasma concentration of moxidectin solution following subcutaneous injection exceeded the linear range. The accuracy and precision (*n* = 6) of the 10-fold diluted rat plasma samples were 96.0% and 7.2%, respectively (Table S1).

### 2.3. Pharmacokinetic Studies

**Figure 3:**
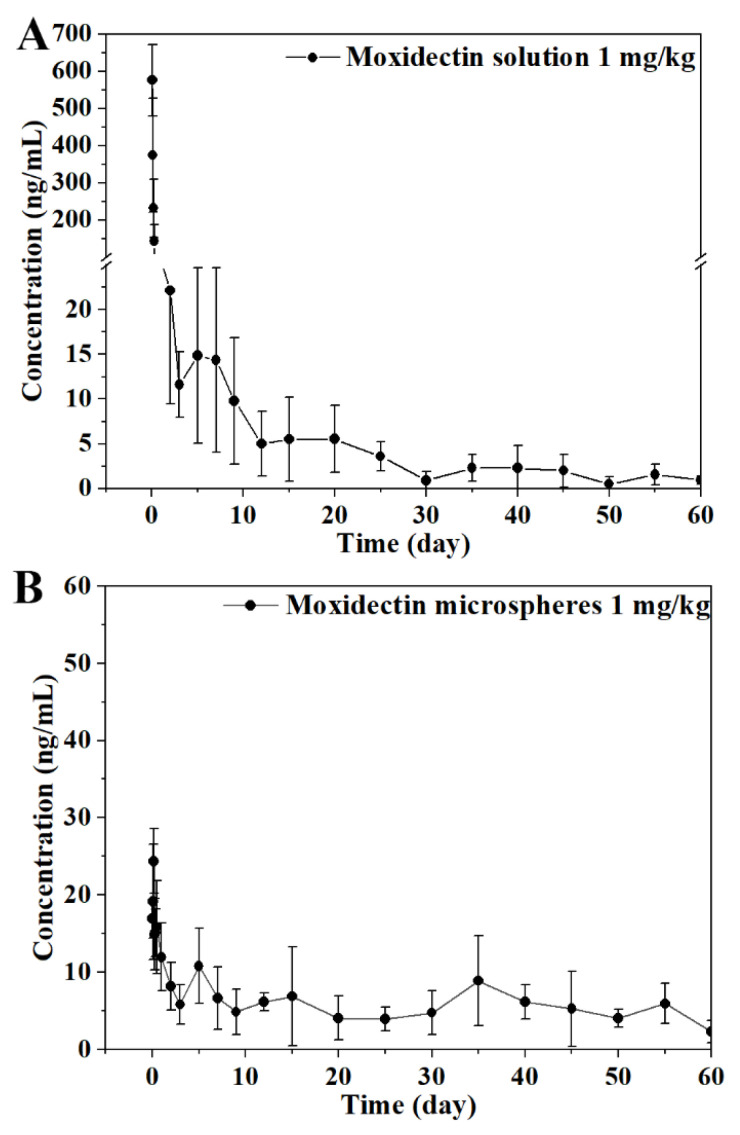
Plasma concentration–time curves of moxidectin in rats after subcutaneous administration (1 mg/kg) (*n* = 6). (**A**) Moxidectin solution; (**B**) Moxidectin microspheres.

The plasma concentration of moxidectin was determined using the validated UHPLC-MS/MS method after subcutaneous injection of MOX-MS (1 mg/kg) or moxidectin solution (1 mg/kg), respectively. The mean plasma concentration–time curves of moxidectin and the main pharmacokinetic parameters of moxidectin are listed (Figure 3 and Table 5). After subcutaneous administration of moxidectin solution at a single dose of 1 mg/kg, the C_max_ of moxidectin in rats was 575.51 ± 96.44 ng/mL, T_max_ was 0.04 ± 0.00 h, T_1/2_ was 8.79 ± 2.21 d, AUC_0–t_ was 418.08 ± 82.31 ng/mL·d, and AUC_0–∞_ was 430.06 ± 86.45 ng/mL·d. In addition, after subcutaneous administration of MOX-MS, the C_max_ of moxidectin in rats was 28.10 ± 4.91 ng/mL, T_max_ was 1.29 ± 2.80 d, T_1/2_ was 25.84 ± 15.21 d, AUC_0–t_ was 376.43 ± 119.19 ng/mL·d, and AUC_0–∞_ was 502.81 ± 178.73 ng/mL·d.

## 3. Discussion

To improve analytical efficiency, it is critical to develop a fast, economical, and reliable sample preparation method for biological samples. Several extraction methods, including protein precipitation (PPT), liquid–liquid extraction (LLE), and solid-phase extraction (SPE) have been widely used in biological sample processing. The PPT method is prone to interference in mass spectrometry ionization, resulting in a severe matrix effect. The SPE method is cumbersome, time-consuming, and expensive [20,21]. Hence, we conducted the current study by using a simple, low matrix effect, and low-cost liquid–liquid extraction method in which only 100 μL of plasma were used and a LLOQ of 1.00 ng/mL was achieved. Acetonitrile was chosen as the extraction solvent due to its higher recovery rate compared to methanol. Furthermore, no SPE columns were used in this study, which significantly reduced the assay costs.

In order to achieve good separation of analytes and IS, high detection sensitivity, and ideal peak shapes, it is very important to optimize UHPLC-MS/MS detection conditions. Because of a better response of moxidectin and IS in the ESI positive mode than that in the ESI negative mode, we chose the ESI positive mode for detection in this study. A mobile phase consisting of a 10 mM ammonium formate solution with 0.1% formic acid (A) and acetonitrile (B) was used to elute moxidectin and IS. Formic acid was added to suppress solvent ionization to obtain the symmetrical peak, whereas ammonium formate was added to enhance the ionization of moxidectin and IS to improve detection sensitivity. Compared to the previously reported methods, an isocratic elution was applied and a shorter detection time was achieved in our study [12,17,22,23], resulting in more concise and shorter run times.

To ensure the accuracy and reliability of the detection results, it is essential to carry out methodological validation. Selectivity, LLOQ, linearity, matrix effect, accuracy, precision, recovery, and stability were evaluated according to the guidance of ICH M10. According to our newly established UHPLC-MS/MS method, the retention times of moxidectin and IS were 2.24 min and 1.88 min, respectively, and there were no interference peaks. A good linear relationship between the concentration ratio of moxidectin and IS and the peak area ratio of moxidectin and IS was established. The intra- and inter-day accuracy and precision of the analysis method met the acceptance criteria. In addition, the stability of moxidectin was demonstrated under various storage conditions. Thus, a sensitive, fast, economical, and reliable UHPLC-MS/MS method was quantified to detect the moxidectin concentration in rat plasma.

Many studies have focused on the tissue residue of moxidectin, but there are limited studies on the pharmacokinetics of moxidectin, especially in rats [24,25,26,27]. In this study, the pharmacokinetics of MOX-MS and moxidectin solution in rats were evaluated to investigate the sustained-release properties of MOX-MS. After a single dose of subcutaneous injection, for the moxidectin solution group, the moxidectin concentration in plasma quickly reached 575.51 ± 96.44 ng/mL in 0.04 ± 0.00 h, while for the MOX-MS group, the moxidectin concentration in plasma only reached 28.10 ± 4.91 ng/mL in 1.29 ± 2.80 d (Figure 3). It is indicated that MOX-MS reduced the C_max_ of moxidectin, which is beneficial for drug safety. The half-life of MOX-MS (T_1/2_ = 25.84 ± 15.21 d) was significantly longer than that of the moxidectin solution group (T_1/2_ = 8.79 ± 2.21 d), suggesting that MOX-MS showed a good sustained-release effect, which was achieved by the slow degradation of polymer microspheres subcutaneously. In addition, the long half-life may be attributed to the high fat distribution of moxidectin. The AUC_0–t_ of MOX-MS is similar to that of the moxidectin solution. However, the AUC_0–∞_ of MOX-MS (502.81 ± 178.73 ng/mL·d) was 1.2 times higher than that of the moxidectin solution (430.06 ± 86.45 ng/mL·d). This might be explained by the fact that C_60d_ (2.3 ng/mL vs. 0.9 ng/mL) and T_1/2_ (25.84 d vs. 8.79 d) of MOX-MS are higher than those of the moxidectin solution, which resulted in the continuous release of the drug from MOX-MS in the third month, while the drug in the moxidectin solution had already been completely metabolized. Therefore, the bioavailability of MOX-MS is actually higher than that of the moxidectin solution. On the other hand, the influence of rat weight, age, and gender on the PK results, which will affect the activity of enzymes and lead to changes in drug absorption and metabolism, cannot be ignored. In summary, MOX-MS showed good sustained-release properties in rats, which may provide data support for the further formulation optimization of MOX-MS.

Although MOX-MS achieved a good release behavior in rats, the pharmacokinetic difference between rats and dogs should be noted due to the fact that there are obvious differences in physiological structure, metabolic pathways, and excretion between dogs and rats. It is reported that AUC_0–t_ and C_max_ increased in a dose-proportional manner from 0.5 mg/kg to 2.5 mg/kg in dogs [28]. And it showed that C_max_ of dogs after subcutaneous administration of 1.5 mg/kg is equivalent to that of rats after subcutaneous administration of 1 mg/kg. That is, drugs are absorbed and metabolized faster in rats than in dogs. Also, as a pharmacokinetic difference in moxidectin in male and female beagle dogs was reported, the pharmacokinetic studies of MOX-MS in male and female dogs, the target animal, should be conducted in our further study [29].

## 4. Materials and Methods

### 4.1. Chemical Materials and Reagents

Standard moxidectin and avermectin B1a (internal standard, IS) were purchased from Stanford Analytical Chemicals Inc. (Denver, CO, USA) with purities of 98.15% and 97.45%, respectively. Blank Sprague-Dawley rat plasma was obtained from iPhase Biosciences Inc. (Suzhou, China). Heparin sodium was purchased from Shanghai yuanye Bio-Technology Co. (Shanghai, China). UHPLC-MS grade acetonitrile (ACN), ammonium formate and formic acid (FA) were provided by Fisher Scientific Co. (Waltham, MA, USA). Water was purified using an ultra-pure water system before use (Water Purifier, Chengdu, China). All other reagents and chemicals were of analytical grade.

### 4.2. UHPLC-MS/MS Conditions

The analyses were performed using an UHPLC-MS/MS system (AB SCIEX QTRAP 5500), which consisted of an ExionLCTM UHPLC system with a binary pump and a triple-quadrupole mass spectrometer with an ESI interface. Avermectin B1a was used as an internal standard (IS) [19]. Samples were separated using an Agilent Zorbax Eclipse Plus C18 column (50 mm × 2.1 mm, 1.8 μm, Agilent, Santa Clara, CA, USA) with a mobile phase consisting of a 10 mM ammonium formate solution with 0.1% formic acid (A) and acetonitrile (B). Mobile phase A and mobile phase B (*v*/*v* 10:90) were isocratically eluted and the flow rate was 0.4 mL/min. The sample was injected at a volume of 10 μL with a column temperature of 35 °C and the running time was set to 5 min.

For all analyses, the mass spectrometer was operated in a positive ion mode. The MS/MS parameters were set as follows: curtain gas was 20 psi, collision gas was medium, ion spray voltage was 5.5 kV, source temperature was 500 °C, ion source gas 1 was 45 psi, and ion source gas 2 was 55 psi. The optimized mass spectrometric parameters were obtained as follows: for moxidectin, the collision energy (CE) was 14 V, and the declustering potential (DP) was 68 V for transitions *m*/*z* 640.5 [M + H]^+^ → 528.4. For avermectin B1a, the collision energy (CE) was 36 V and the declustering potential (DP) was 61 V for transitions *m*/*z* 890.7 [M + NH_4_]^+^ → 305.3. The SCIEX OS software (Version 2.1.6.59781, Danaher, Framingham, MA, USA) was used for the data analysis.

### 4.3. Preparation of Standard Curve and Quality Control (QC) Samples

The stock solution of moxidectin was prepared by placing 5.0 mg of moxidectin into a 25 mL brown volumetric flask and dissolving it in ACN to a final concentration of 200 μg/mL. The standard working solutions were prepared by diluting the stock solution of moxidectin using ACN. Calibration standards were prepared by mixing 10 μL of working solution with 90 μL of blank rat plasma to achieve a final concentration of 1.00, 2.00, 5.00, 10.0, 20.0, 50.0, 100, and 200 ng/mL. Similarly, QC samples with concentrations of 1.00 ng/mL (LLOQ), 5.00 ng/mL (LQC), 50.0 ng/mL (MQC), and 150 ng/mL (HQC) were prepared using the corresponding standard working solution and blank rat plasma. The stock solution of IS was prepared by placing 10.0 mg of avermectin B1a into a 100 mL brown volumetric flask and dissolving it in ACN to a final concentration of 100 μg/mL. The working solution of IS (200 ng/mL) was prepared by diluting its stock solution using ACN. All solutions were stored at −20 °C for further use.

### 4.4. Sample Preparation

To 100 μL of plasma, a solution of IS (200 ng/mL, 10 μL) was added and vortexed for 30 s. Then, 2 mL of ACN were added to the mixture and vortexed for 5 min. The supernatant was taken into a 4 mL centrifuge tube after centrifuging at 12,000 rpm (13,400× *g*) for 10 min at 4 °C. Subsequently, the supernatant was evaporated at 40 °C to dry, and the residue was dissolved using 100 μL of mobile phase A and mobile phase B (*v*/*v* 10:90) and vortexed for 10 min. Finally, 10 μL of the supernatant was subjected to UHPLC-MS/MS analysis after centrifuging at 12,000 rpm (13,400× *g*) for 10 min at 4 °C [30].

### 4.5. Method Validation

The UHPLC-MS/MS validation was carried out in terms of selectivity, LLOQ, linearity, matrix effect, accuracy, precision, recovery, and stability according to the ICH harmonized Guideline, Bioanalytical Method Validation and Study Sample Analysis (M10) [31,32].

#### 4.5.1. Selectivity and Matrix Effect

The selectivity of the analytical methods was evaluated by comparing the chromatograms of six different batches of rat plasma with those of corresponding plasma samples spiked with moxidectin and IS to confirm that they were free of the interfering peaks [33].

The matrix effect was determined by comparing the peak area ratios of post-extraction blank plasma samples spiked with moxidectin at three QC levels with standard solutions of equivalent concentrations that were dried directly and reconstituted with the same mobile phase. Also, to investigate the effect of microspheres on the matrix effect, the peak area ratios of blank microsphere plasma spiked with moxidectin at three QC levels with standard solutions of equivalent concentrations were compared.

#### 4.5.2. LLOQ and Linearity

The lower limit of detection (LLOD) and the lower limit of quantification (LLOQ) were determined as the ratios of signal to baseline noise (S/N) of at least 3 and 10, respectively.

A double blank (no analyte, no IS), a blank sample (blank plus IS), and at least six calibrator samples (ranging from 1.00 to 200 ng/mL) were prepared to determine the calibration curve. The calibration curve was obtained by a linear regression of the concentration ratio of moxidectin to IS against the peak area ratio of moxidectin to IS and weighted by 1/x. To meet the criteria, a correlation coefficient (r^2^) of at least 0.99 is required, and a minimum of six non-zero calibration levels should fulfill the criteria of accuracy.

#### 4.5.3. Accuracy and Precision

The accuracy and precision (A & P) of the UHPLC-MS/MS method were established with three independent runs, four QC levels (LLOQ, LQC, MQC and HQC) per run, and six replicates per QC level. The accuracy was defined as the ratio of measured and theoretical concentrations, and the precision was defined as relative standard deviation (RSD) of repeated measures. The intra-day A & P were evaluated by analyzing four QC concentrations (1.00 ng/mL, 5.00 ng/mL, 50.0 ng/mL, 150 ng/mL) in six replicates per QC level on the same day. The inter-day A & P were evaluated by analyzing four QC concentrations in six replicates per QC level over three days. The accepted criteria for A & P were ±15%, except ±20% at LLOQ.

#### 4.5.4. Recovery and Stability

The extraction recovery was determined by comparing the analytical results of extracted samples with the extracts of blanks spiked with the analyte post-extraction at three QC levels (LQC, MQC and HQC, *n* = 6). The extraction recovery for all analytes and the internal standard should be consistent.

The stability was evaluated by analyzing QC samples at L, M, and H concentrations (*n* = 6) under different storage conditions: (1) QC samples were kept at ambient temperature for 24 h; (2) QC samples were stored at −20 °C for 60 days (long-term stability); (3) QC samples were kept in the auto-sampler for 24 h at 4 °C; (4) QC samples have undergone three complete freeze–thaw cycles (−20 °C to room temperature as one cycle).

#### 4.5.5. Dilution Integrity

To determine whether moxidectin was accurately quantified at concentrations above the maximum values of the calibration curve, the dilution integrity of moxidectin was examined. Six aliquots of moxidectin (1000 ng/mL) rat plasma were diluted 10-fold with blank rat plasma. The diluted samples were further quantified according to the calibration curve. The acceptance criteria for accuracy and precision of the diluted samples should be within the acceptable limit (RSD ≤ 15%).

### 4.6. Pharmacokinetic Study

Twelve male Sprague-Dawley rats with a body weight of 200 to 220 g were purchased from the Lanzhou Veterinary Research Institute of Chinese Academy of Agriculture Science and housed in the standard environmentally controlled animal room (temperature: 25 ± 2 °C, relative humidity: 50%, 12:12 h light/dark cycle) for one week before the experiment. All rats were maintained under almost the same conditions, with free access to food and water. The experiments were carried out according to the ARRIVE guidelines and approved by the Ethics Committee of Lanzhou Institute of Husbandry and Pharmaceutical Science of Chinese Academy of Agricultural Sciences (SYXK-2023-005) (Lanzhou, China). During the experiment, all precautions were taken to minimize animal suffering.

The moxidectin solution was prepared by dissolving 40 mg of moxidectin in 100 mL of 1% Tween 80. The freeze-dried powder of MOX-MS was dispersed in an aqueous medium that consisted of 0.87% sodium chloride, 0.75% sodium carboxymethyl cellulose, and 0.1% Tween 20 [34]. All rats were randomly divided into two groups, including the moxidectin solution group and the MOX-MS group (*n* = 6). Two groups of rats were subcutaneously injected with the moxidectin solution and MOX-MS at a single dose of 1 mg/kg, respectively [13,15]. Blood samples (0.3 mL) were collected through the orbital vein at 0 h, 1 h, 2 h, 4 h, 6 h, 8 h, 12 h, 1 d, 2 d, 3 d, 5 d, 7 d, 9 d, 12 d, 15 d, 20 d, 25 d, 30 d, 35 d, 40 d, 45 d, 50 d, 55 d and 60 d. All samples were centrifuged at 3000 rpm for 10 min at 4 °C. Plasma samples were collected and stored at −20 °C until the UHPLC-MS/MS analysis.

### 4.7. Data Analysis

Plasma concentrations of moxidectin were determined using the established method, and the pharmacokinetic parameters were calculated using a non-compartmental model by PK Solver software (version 2.0, China Pharmaceutical University, China). All results were expressed as the mean ± standard deviation (SD).

## 5. Conclusions

A sensitive, economical, and reliable UHPLC-MS/MS method was developed in this study to determine moxidectin levels in rats after subcutaneous administration. Compared with the reported analytical method, our developed analytical method significantly shortened run times, simplified sample processing steps, and saved costs. It was the first method used for moxidectin detection in rat plasma without additional devices (Mitra^®^), which is validated in terms of sensitivity, linearity, recovery, matrix effect, accuracy, precision, and stability. Moreover, the established method was successfully applied to evaluate the pharmacokinetics of MOX-MS in rats, which may accelerate the development of moxidectin formulations.

## Figures and Tables

**Table 1:** Recovery and matrix effect of moxidectin in rat plasma (*n* = 6).

Concentration (ng/mL)	Recovery (%)		Matrix Effects (%)			
	Mean ± SD	RSD (%)	Mean ± SD ^a^	RSD ^a^ (%)	Mean ± SD ^b^	RSD ^b^ (%)
5.00	94.4 ± 3.8	4.0	94.8 ± 4.4	4.7	91.2 ± 8.7	9.6
50.0	94.1 ± 4.8	5.1	96.2 ± 5.0	5.2	94.4 ± 8.3	8.6
150	98.0 ± 4.7	4.8	94.7 ± 5.7	6.1	95.0 ± 3.4	4.2

^a^ Matrix effect of blank plasma. ^b^ Matrix effect of blank plasma in the presence of blank microspheres.

**Table 2:** Calibration curve parameters for moxidectin in rat plasma (*n* = 3).

Sample Matrix	Calibration Range (ng/mL)	Slope	Intercept	r^2^
		Mean ± SD	Mean ± SD	Mean ± SD
Rat plasma	1.00–200	0.0277 ± 0.0088	0.0659 ± 0.0441	0.9970 ± 0.0022

**Table 3:** Intra- and inter-day precision and accuracy of the determination of moxidectin in rat plasma.

Concentration (ng/mL)	Intra-Day Precision and Accuracy (*n* = 6)		Inter-Day Precision and Accuracy (*n* = 18)	
	Accuracy (%) ± SD	RSD (%)	Accuracy (%) ± SD	RSD (%)
1.00	103.6 ± 0.1	7.2	103.0 ± 0.1	6.4
5.00	100.3 ± 0.2	4.1	100.1 ± 0.3	5.4
50.0	103.2 ± 3.1	6.0	100.7 ± 2.9	5.8
150	101.3 ± 4.0	2.6	101.8 ± 6.7	4.4

**Table 4:** Stability results of moxidectin in rat plasma samples under various conditions (*n* = 6).

Storage Conditions	Concentration (ng/mL)	Accuracy ± SD (%)	RSD (%)
Ambient temperature for 24 h	5.00	99.6 ± 0.0	0.4
	50.0	98.3 ± 0.4	0.8
	150	100.6 ± 2.6	1.7
At −20 °C for 60 days	5.00	101.4 ± 0.1	2.2
	50.0	103.2 ± 1.2	2.2
	150	100.4 ± 1.3	0.9
At 4 °C in the autosampler for 24 h	5.00	99.8 ± 0.1	2.1
	50.0	100.6 ± 2.3	4.6
	150	100.6 ± 1.5	1.0
Three freeze–thaw cycles	5.00	105.7 ± 0.2	3.9
	50.0	100.8 ± 1.3	2.5
	150	102.1 ± 2.7	1.8

**Table 5:** The pharmacokinetic parameters of moxidectin in rat after subcutaneous administration (*n* = 6, Mean ± SD).

Pharmacokinetic Parameter	Moxidectin Solution	Moxidectin Microspheres
T_1/2_ (d)	8.79 ± 2.21	25.84 ± 15.21
T_max_ (d)	0.04 ± 0.00	1.29 ± 2.80
C_max_ (ng/mL)	575.51 ± 96.44	28.10 ± 4.91
AUC_0–t_ (ng/mL·d)	418.08 ± 82.31	376.43 ± 119.19
AUC_0–∞_ (ng/mL·d)	430.06 ± 86.45	502.81 ± 178.73
MRT_0–∞_ (d)	12.45 ± 3.05	46.13 ± 15.03

## Data Availability

The data presented in this study are available on request from the corresponding author.

## Supplementary Materials

The following supporting information can be downloaded at: https://www.mdpi.com/article/10.3390/molecules29204786/s1, Figure S1: Q1MS spectra of moxidectin (IS); Figure S2: Q1MS spectra of avermectin B1a; Figure S3: Calibration curve; Figure S4: Weight of rats during the PK study; Table S1: Precision and Accuracy after dilution for moxidectin (1:10).
