# Supplementary material for: Development of a UHPLC-MS/MS Method for the Determination of Moxidectin in Rat Plasma and Its Application in Pharmacokinetics
Source: Molecules. 2024 Oct 10;29(20):4786. doi: 10.3390/molecules29204786 (PMC11510379; doi:10.3390/molecules29204786)
Supplement: Supplementary file 1 [file molecules-29-04786-s001.zip › molecules-3230196-supplementary.pdf]

Supplementary Material

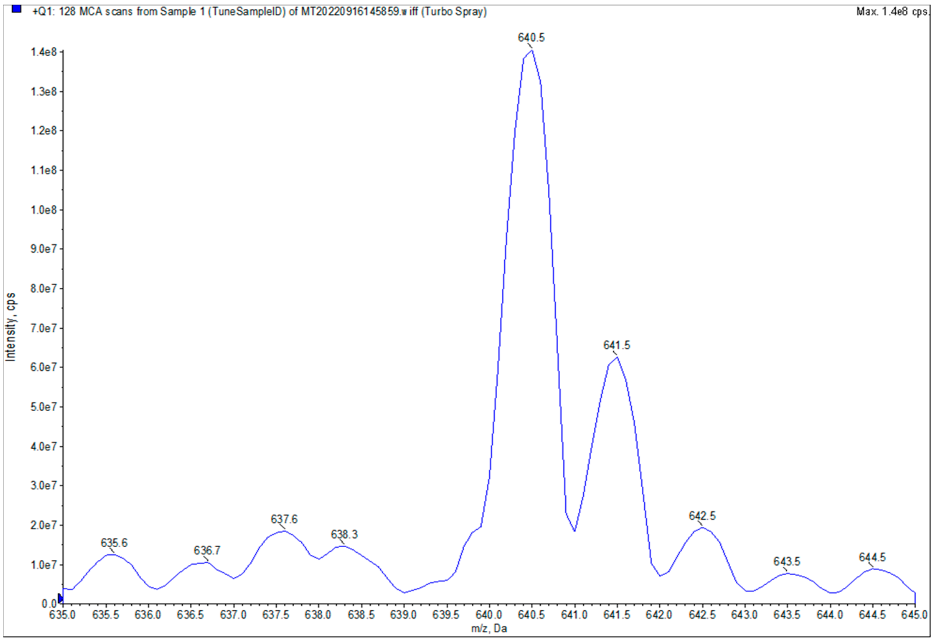

Figure S1. Q1MS spectra of moxidectin

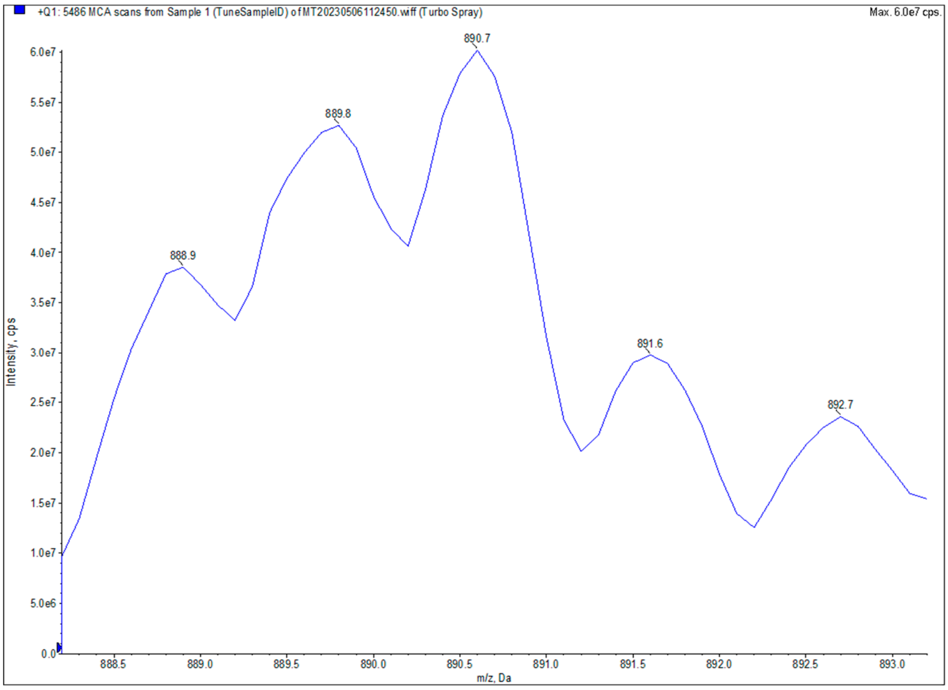

Figure S2. Q1MS spectra of avermectin B1a (IS)

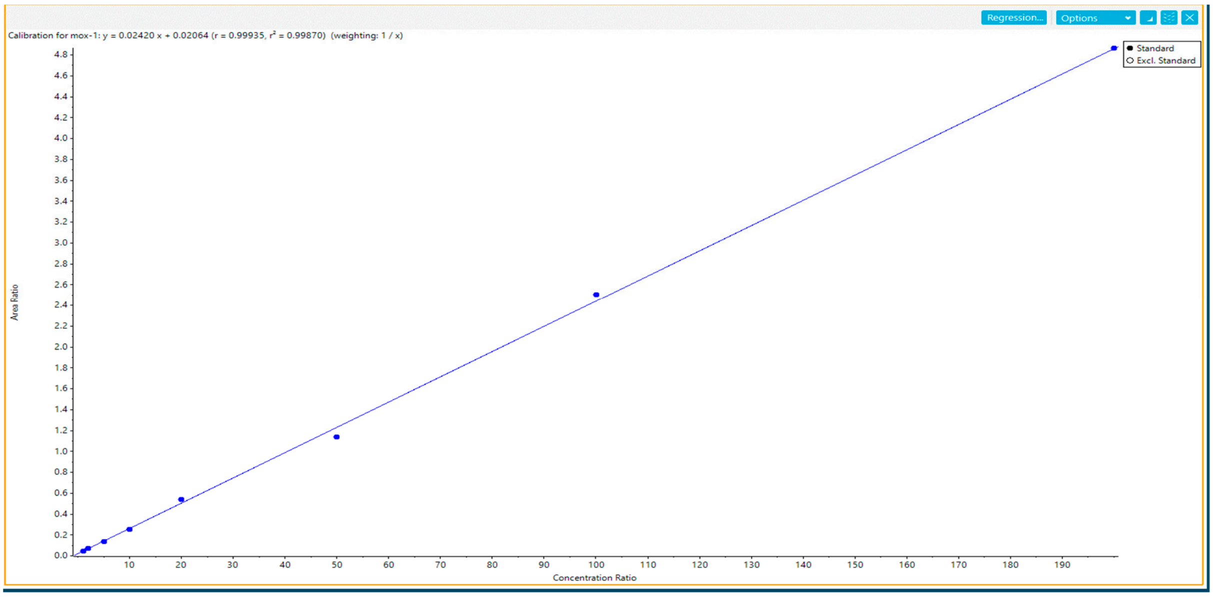

Figure S3. Calibration Curve

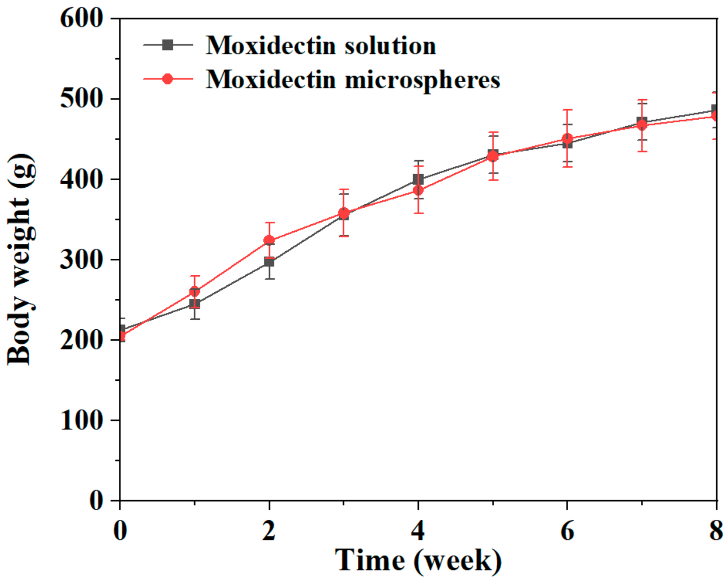

Figure S4. Weight of rats during the PK study

Table S1 Precision and Accuracy after dilution for moxidectin (1:10)

1	2	3	4	5	6	Accuracy ± SD (%)	RSD
88.0	102.0	92.8	102.5	88.7	101.8	96.0 ± 6.9	7.2
